# Supplementary material for: Revisiting Multi-Omics Data to Unravel Galectins as Prognostic Factors in Head and Neck Squamous Cell Carcinoma
Source: Biomedicines. 2024 Feb 27;12(3):529. doi: 10.3390/biomedicines12030529 (PMC10968280; doi:10.3390/biomedicines12030529)
Supplement: Supplementary file 1 [file biomedicines-12-00529-s001.zip › biomedicines-2844055-supplementary.pdf]

Supplementary Material

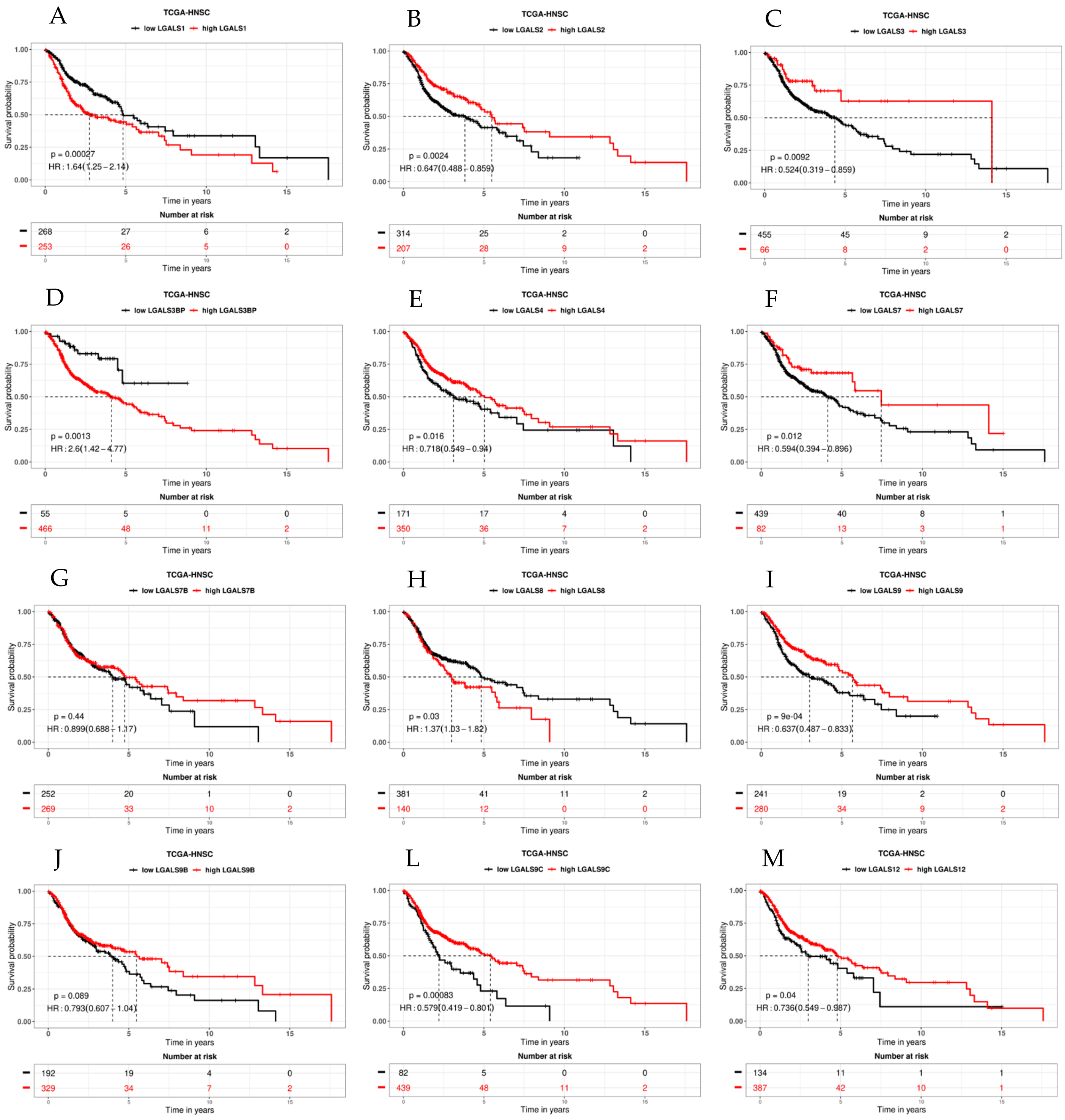

Supplementary Figure S1: Survival analysis in ToPP Database. Kaplan-Meyer curves for patient prognosis when divided by of LGALS1 (A), LGALS2 (B), LGALS3 (C), LGAL3BP (D), LGALS4 (E), LGALS7 (F), LGALS7B (G), LGALS8 (H), LGALS9 (I), LGALS9B (J), LGALS9C (L) and LGALS12 (M) expression levels. Black data points represent patients whose HNSCC tumors had a galectin expression below the median level. Red data points represent patients whose HNSCC tumors had a galectin expression above the median level.

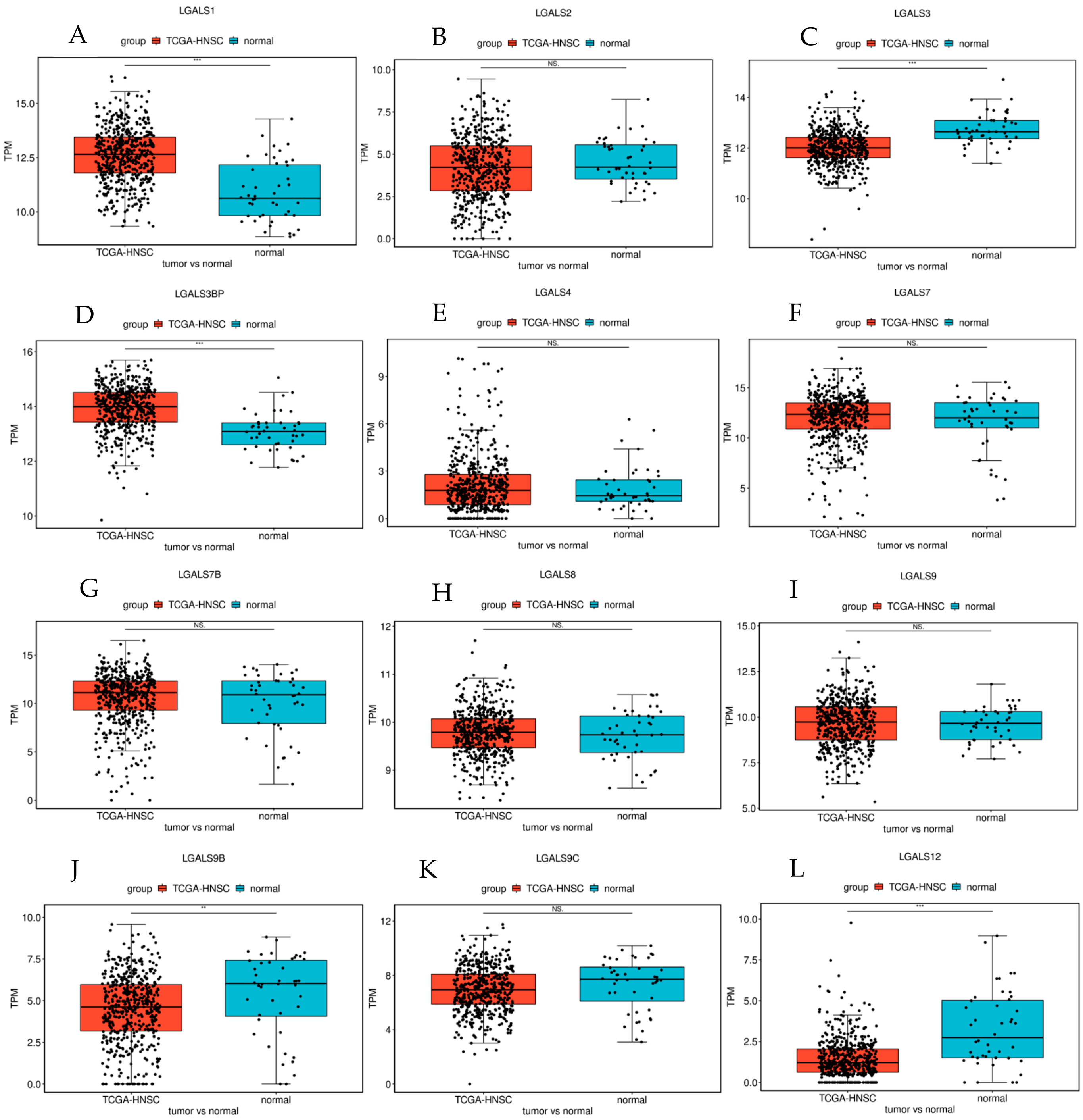

Supplementary Figure S2: Differential expression of galectins in HNSCC patients and healthy individuals in ToPP using TCGA dataset. Box plot of expression of LGALS1 (A), LGALS2 (B), LGALS3 (C), LGAL3BP (D), LGALS4 (E), LGALS7 (F), LGALS7B (G), LGALS8 (H), LGALS9 (I), LGALS9B (J), LGALS9C (K) and LGALS12 (L) in HNSCC (red) and healthy tissue samples (blue). Legend: *, $P < 0.05$; **; $P < 0.01$; ***, $P < 0.001$; NS, not significant.

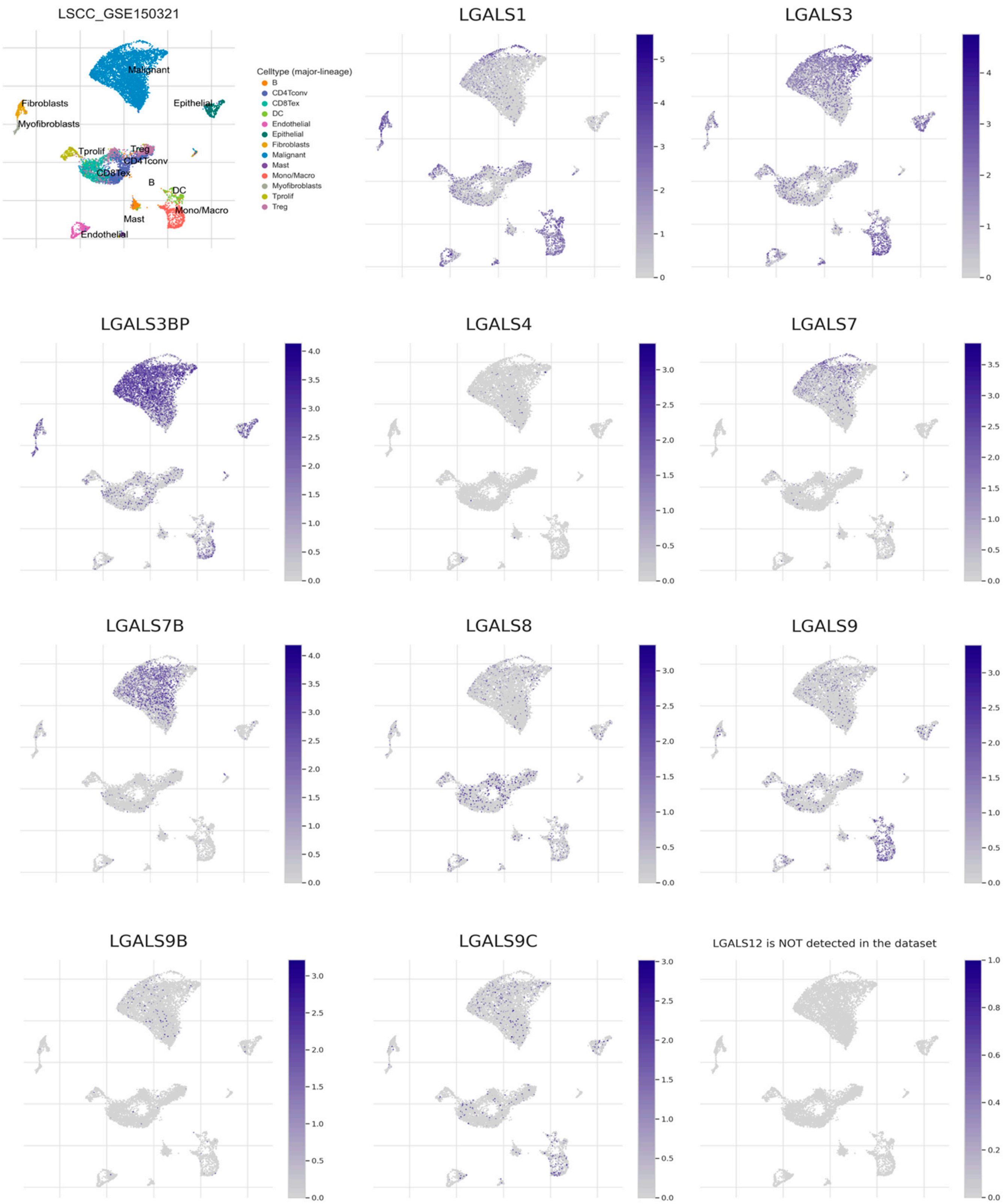

Supplementary Figure S3: Expression of galectins in GSE150321 dataset in TISCH2. The color intensity is proportional to the expression of each of the galectins of interest.

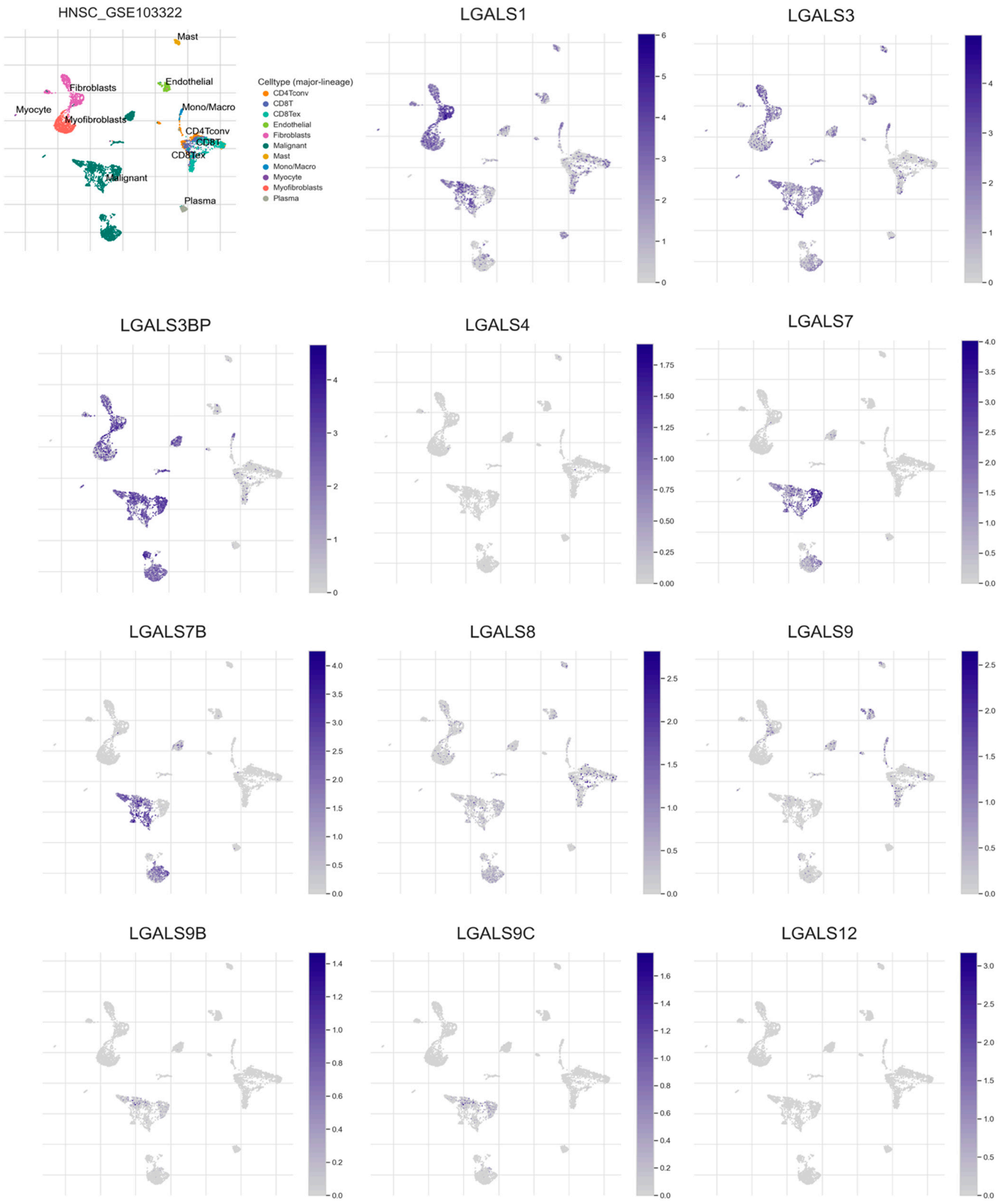

Supplementary Figure S4: Expression of galectins in GSE103322 dataset in TISCH2. The color intensity is proportional to the expression of each of the galectins of interest.

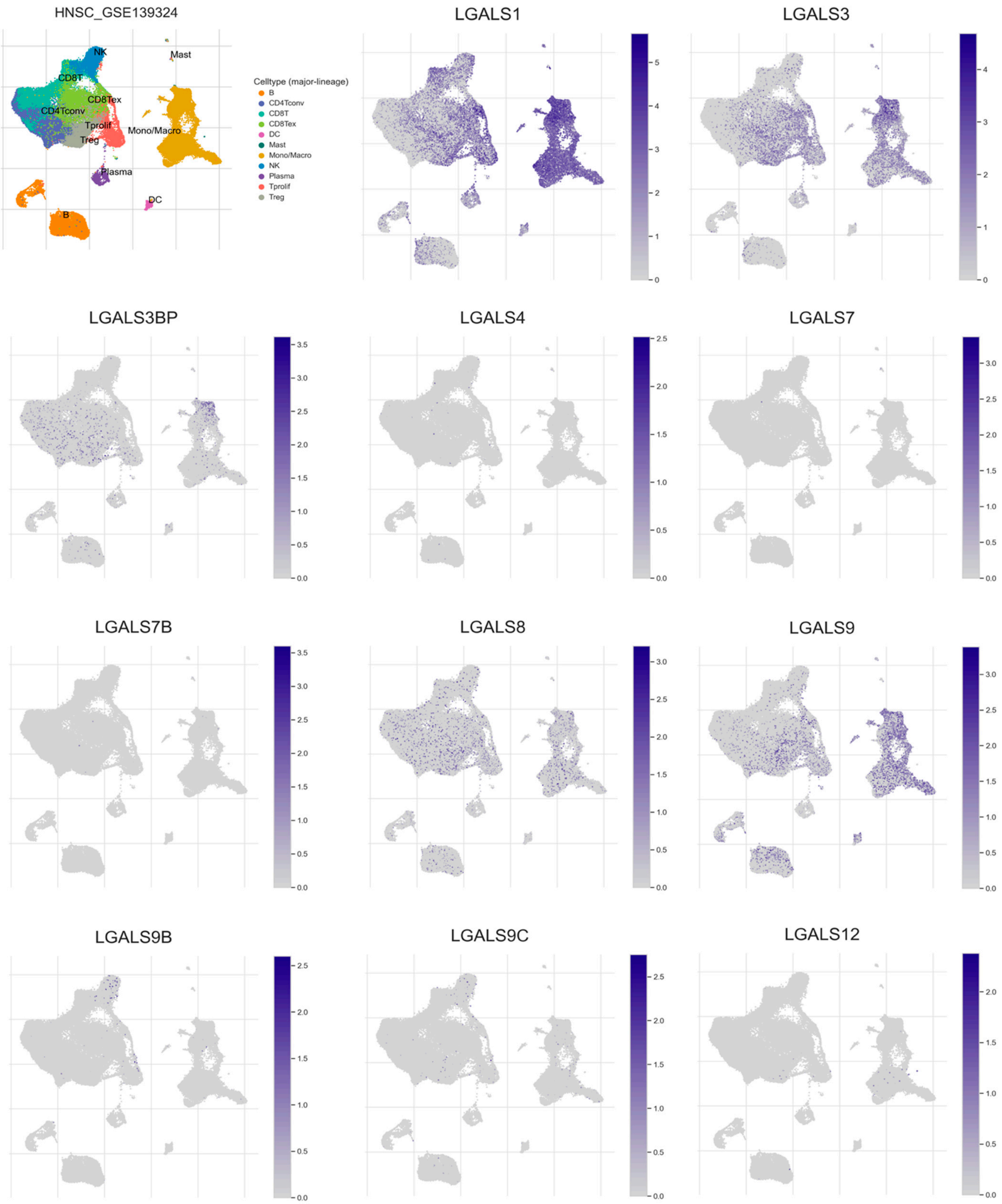

Supplementary Figure S5: Expression of galectins in GSE139324 dataset in TISCH2. The color intensity is proportional to the expression of each of the galectins of interest.

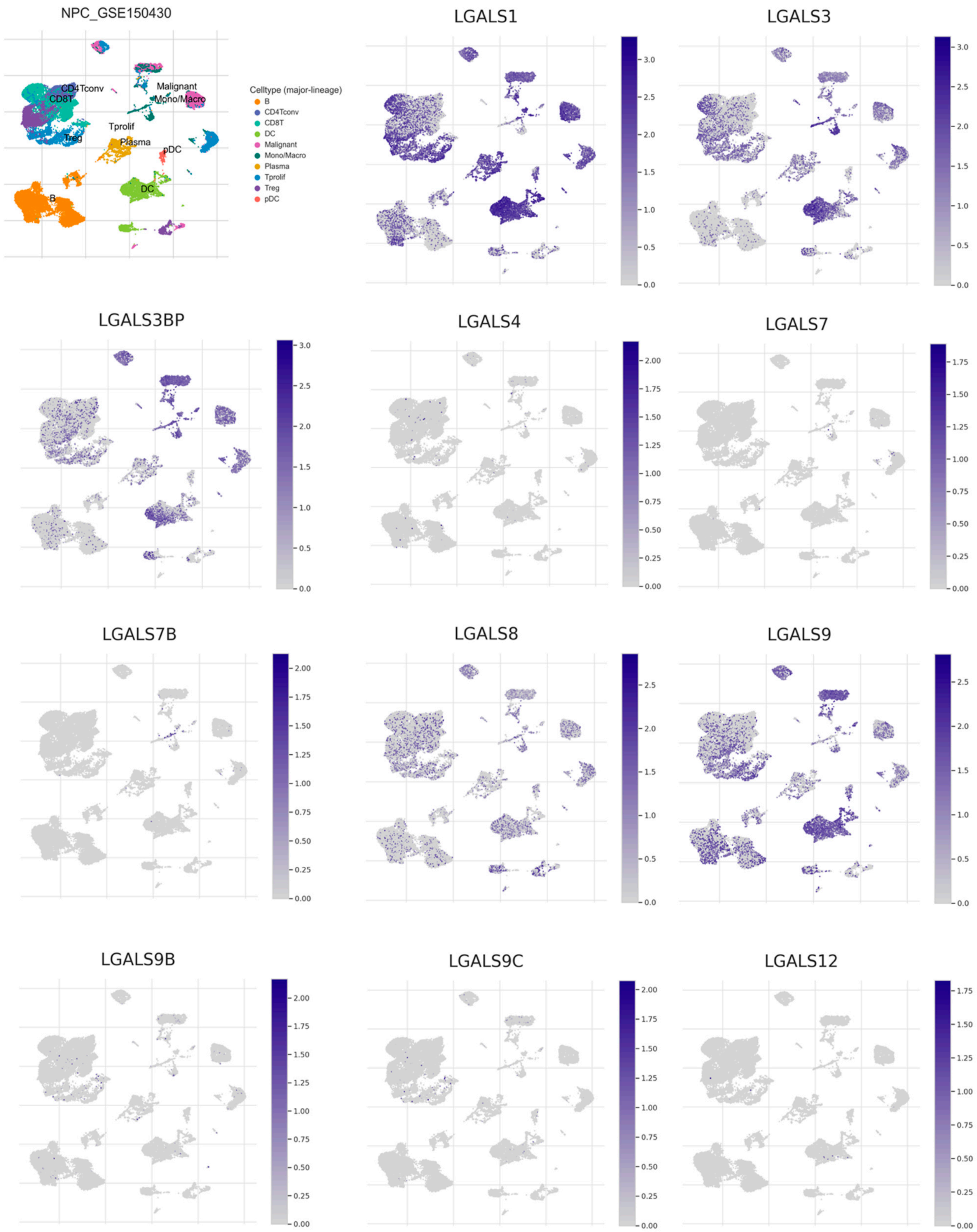

Supplementary Figure S6: Expression of galectins in GSE150430 dataset in TISCH2. The color intensity is proportional to the expression of each of the galectins of interest.

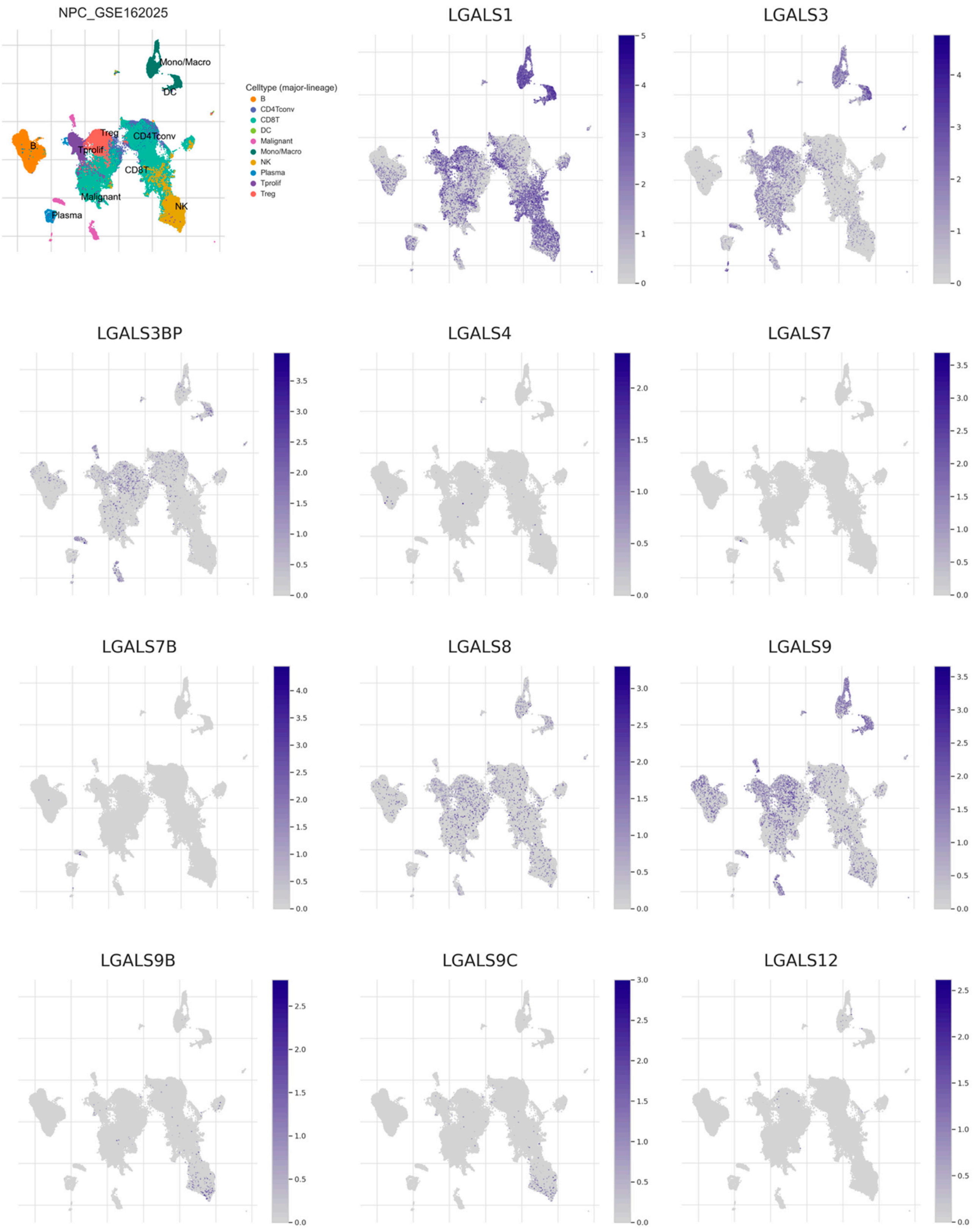

Supplementary Figure S7: Expression of galectins in GSE162025 dataset in TISCH2. The color intensity is proportional to the expression of each of the galectins of interest.

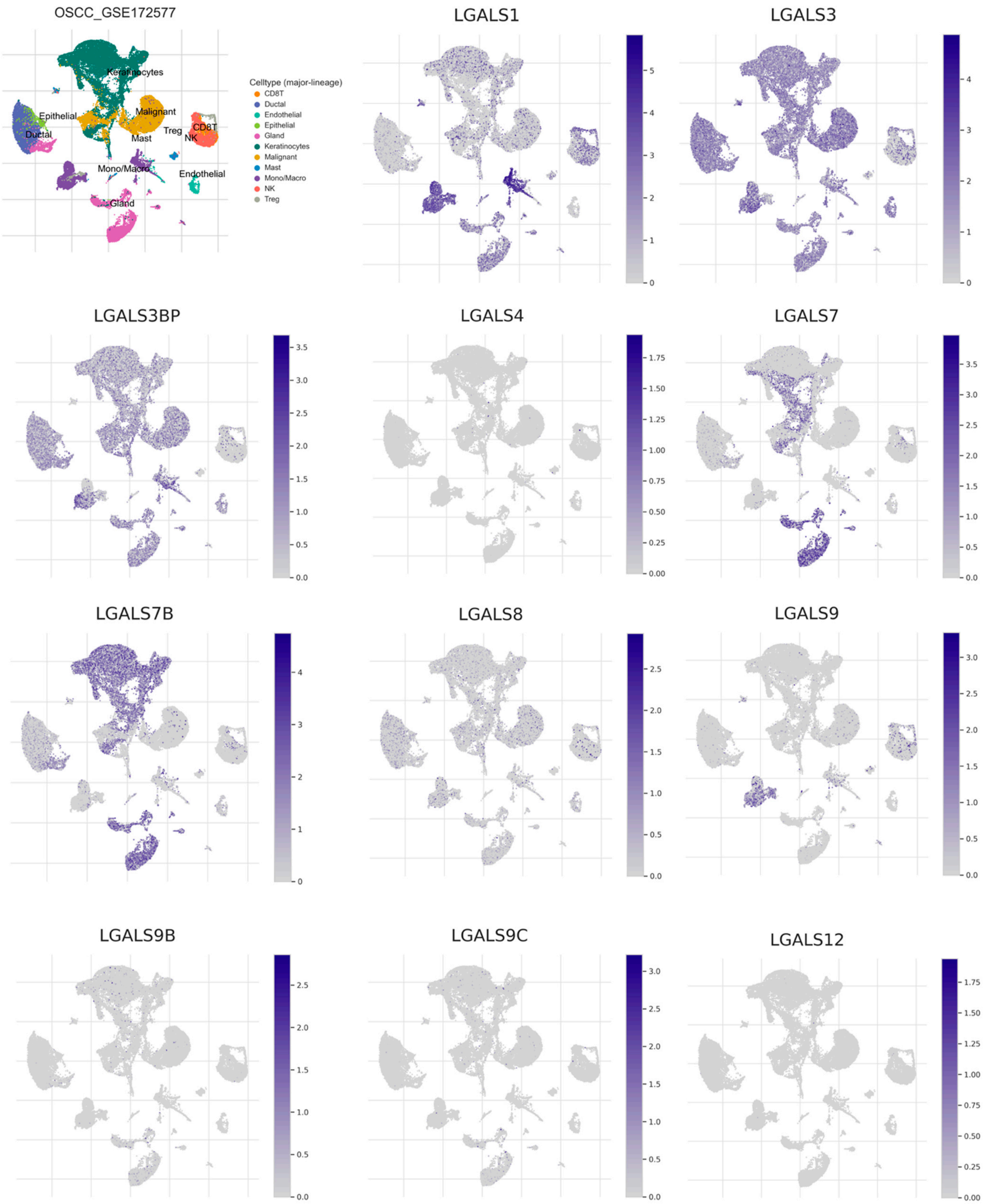

Supplementary Figure S8: Expression of galectins in GSE172577 dataset in TISCH2. The color intensity is proportional to the expression of each of the galectins of interest.

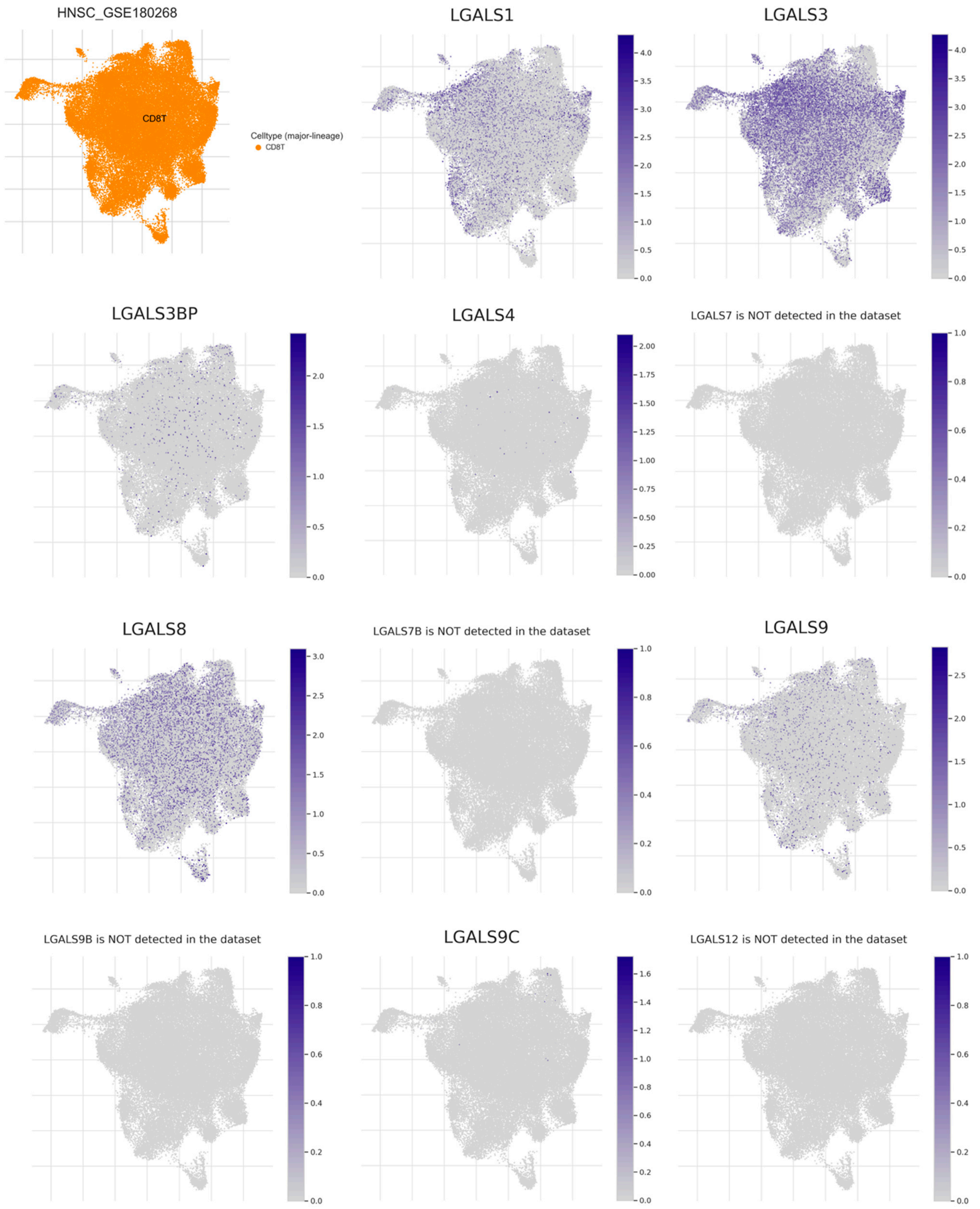

Supplementary Figure S9: Expression of galectins in GSE180268 dataset in TISCH2. The color intensity is proportional to the expression of each of the galectins of interest.

Cell Type	GEO Datasets	1	3	3BP	4	7	7B	8	9	9B	9C	12
B	LSCC_GSE150321	+	+	+	+/-	+/-	+/-	+	+	+/-	+	ND
	HNSCC_GSE139324	++	+	+/-	+/-	+/-	+/-	+/-	+/-	+/-	+/-	+/-
	NPC_GSE150430	++	+	+	+/-	+/-	+/-	+	++	+/-	+/-	+/-
	NPC_GSE162025	+	+/-	+/-	+/-	+/-	+/-	+/-	++	+/-	+/-	+/-
Tprolif	LSCC_GSE150321	++	+	+	+/-	+/-	+/-	+	+	+/-	+/-	ND
	HNSCC_GSE139324	+++	++	+/-	+/-	+/-	+	+	+/-	+/-	+/-	+/-
	NPC_GSE150430	+++	++	+	+/-	+/-	+/-	+	++	+/-	+/-	+/-
	NPC_GSE162025	+++	++	+/-	+/-	+/-	+/-	+	++	+/-	+/-	+/-
Treg	LSCC_GSE150321	++	++	+	+/-	+/-	+/-	+	+	+/-	+/-	ND
	HNSCC_GSE139324	++	++	+	+/-	+/-	+/-	+	+	+/-	+/-	+/-
	NPC_GSE150430	+++	++	+	+/-	+/-	+/-	+	++	+/-	+/-	+/-
	NPC_GSE162025	+++	++	+	+/-	+/-	+/-	+	++	+/-	+/-	+/-
	OSCC_GSE172577	++	+	+/-	+/-	+/-	+/-	+/-	+	+/-	+/-	+/-
TCD8	LSCC_GSE150321	+	+	+	+/-	+/-	+/-	+	+	+/-	+/-	ND
	HNSCC_GSE103322	+++	++	+	+/-	+/-	++	+	+/-	+/-	+/-	+/-
	HNSCC_GSE139324	++	++	+	+/-	+/-	+/-	+	+	+/-	+/-	+/-
	NPC_GSE150430	+++	++	+	+/-	+/-	+/-	+	+	+/-	+/-	+/-
	NPC_GSE162025	+++	++	+	+/-	+/-	+/-	+	+	+/-	+/-	+/-
	OSCC_GSE172577	++	+	+/-	+/-	+/-	+	+	+	+/-	+/-	+/-
	HNSCC_GSE180268	++	+++	+	+/-	+/-	ND	++	+	ND	+/-	ND
TCD4	LSCC_GSE150321	+	+	+	+/-	+/-	+/-	+	+	+/-	+	ND
	HNSCC_GSE103322	+	+/-	+/-	+/-	+/-	+/-	+	+/-	+/-	+/-	+/-
	HNSCC_GSE139324	+	+	+	+/-	+/-	+/-	+	+	+/-	+/-	+/-
	NPC_GSE150430	++	+	+	+/-	+/-	+/-	+	+	+/-	+/-	+/-
	NPC_GSE162025	+	+/-	+	+/-	+/-	+/-	+	+	+/-	+/-	+/-
DC	LSCC_GSE150321	++	+	+/-	+/-	+/-	+/-	+/-	++	+/-	+/-	ND
	HNSCC_GSE139324	+	+/-	+/-	+/-	+/-	+/-	+/-	+	+/-	+/-	+/-
	NPC_GSE150430	+++	+++	++	+/-	+/-	+/-	+	+++	+/-	+/-	+/-
NK cell	HNSCC_GSE139324	++	+	+/-	+/-	+/-	+/-	+	+	+/-	+/-	+/-
	NPC_GSE162025	+++	+	+/-	+/-	+/-	+/-	+	+	+/-	+/-	+/-
	OSCC_GSE172577	+++	+	+/-	+/-	+/-	+/-	+	+	+/-	+/-	+/-
Malignant	LSCC_GSE150321	+	++	+++	+	+	++	+	+	+	+	ND
	HNSCC_GSE103322	+++	+++	+/-	+/-	+/-	+/-	+	+	+/-	+/-	+/-
	NPC_GSE162025	++	+++	+	+/-	+/-	+/-	+/-	++	+/-	+/-	+/-
	OSCC_GSE172577	++	+	+/-	+/-	+/-	+	+	+	+/-	+/-	+/-
Mastocyte	LSCC_GSE150321	+++	+++	+/-	+/-	+/-	+/-	++	+/-	+/-	+	ND
	HNSCC_GSE103322	++	++	+/-	+/-	+/-	+/-	+	+	+/-	+/-	+/-
	OSCC_GSE172577	+	+	+/-	+/-	+/-	+/-	+/-	+	+/-	+/-	+/-
Mono/Macro	LSCC_GSE150321	+++	+++	++	+/-	+/-	+/-	+	+++	+/-	+	ND
	HNSCC_GSE103322	+++	++	+	+/-	+/-	+/-	+/-	++	+/-	+/-	+/-
	HNSCC_GSE139324	+++	++	+	+/-	+/-	+/-	+	++	+/-	+/-	+/-
	NPC_GSE150430	+++	+++	++	+/-	+/-	+/-	+	+	+/-	+/-	+/-
	NPC_GSE162025	+++	++	+/-	+/-	+/-	+/-	+	++	+/-	+/-	+/-
	OSCC_GSE172577	+++	++	++	+/-	+/-	+/-	+/-	++	+/-	+/-	+/-
Myofibroblasts	LSCC_GSE150321	+++	++	+++	+/-	+/-	+	+	+	+/-	+	ND
	HNSCC_GSE103322	+++	++	+++	+/-	+/-	+/-	+/-	+/-	+/-	+/-	+/-
Fibroblasts	LSCC_GSE150321	+++	++	+++	+/-	+/-	+	+	+	+/-	+	ND
	HNSCC_GSE103322	++	++	+++	+/-	++	++	+/-	+/-	+/-	+/-	+/-

Supplementary Figure S10: Expression of galectins in several types of immune cells from HNSCC Dataset using TISCH2. Legend: 1, galectin-1; 3, galectin-3; 3BP, galectin-3BP; 4, galectin-4; 7, galectin-7; 7B, galectin-7B, 8, galectin-8; 9, galectin-9; 9B, galectin-9B; 9C, galectin-9C; 12, galectin-12; B, B cell; DC, dendritic cells; mono/macro, monocyte/macrophage; NK cell; natural killer cell; Tprolif, proliferating T cell; Treg, regulatory T cell.

		1	3	3BP	4	7	7B	8	9	9B	9C	12
Wound Healing (C1)	OS	HR=0.365 (0.18-0.76) ***										HR=0.537 (0.32-0.91) *
	PFI	HR=1.83 (1.05-3.17) *										
	DSS	HR=0.274 (0.12-0.64) **										HR=0.439 (0.23-0.84) *
	DFI				HR=0.04 (0.00-0.51) ***			HR=0.024 (0.01-1.04) *		HR=1.7x10^{9} (0-Inf) *	HR=3.1x10^{9} (0-Inf) *	HR=7.2x10^{9} (0-Inf) ***
	RFS	HR=2.62 (1.17-5.87) *	HR=2.97 (1.02-8.68) *			HR=2.49 (0.99-6.24) *	HR=2.66 (1.06-6.69) *	HR=0.420 (0.19-0.93) *	HR=0.396 (0.15-0.99) *			HR=2.65 (1.10-6.38) *
IFN-γ Dominant (C2)	OS	HR=1.83 (1.28-2.62) ***	HR=0.441 (0.23-0.84) *	HR=2.24 (1.41-3.55) ***	HR=0.641 (0.47-0.88) **	HR=0.576 (0.36-0.91) *		HR=1.61 (1.03-2.51) *	HR=0.575 (0.42-0.79) ***	HR=1.70 (1.10-2.63) *		
	PFI	HR=1.97 (1.36-2.84) ***	HR=0.562 (0.34-0.94) *	HR=1.60 (1.11-2.29) *	HR=0.682 (0.47-0.98) *			HR=1.72 (1.05-2.82) *	HR=0.614 (0.42-0.91) *	HR=0.688 (0.49-0.96) *	HR=0.658 (0.46-0.93) *	
	DSS	HR=2.79 (1.81-4.30) ***	HR=0.322 (0.14-0.74) **	HR=3.26 (1.73-6.14) ***	HR=0.515 (0.34-0.79) **			HR=1.88 (1.00-3.53) *	HR=0.483 (0.32-0.74) ***	HR=0.653 (0.43-0.99) *	HR=0.569 (0.36-0.91) *	
	DFI				HR=0.262 (0.11-0.62) ***	HR=0.358 (0.15-0.83) *	HR=0.43 (0.18-0.99) *		HR=2.62 (1.07-6.44) *	HR=0.421 (0.18-1) *	HR=0.283 (0.12-0.68) **	
	RFS	HR=1.80 (1.12-2.90) *	HR=0.606 (0.383-0.959) *	HR=1.70 (1.07-2.72) *				HR=1.66 (1.07-2.58) *		HR=0.623 (0.40-0.97) *	HR=0.540 (0.35-0.84) **	

Supplementary Figure S11: Survival analysis for each galectin in C1 and C2 immune phenotypes of HNSCC Dataset in ToPP. Abbreviations: *, $P < 0.05$; **; $P < 0.01$; ***, $P < 0.001$; 1, galectin-1; 3, galectin-3; 3BP, galectin-3BP; 4, galectin-4; 7, galectin-7; 7B, galectin-7B, 8, galectin-8; 9, galectin-9; 9B, galectin-9B; 9C, galectin-9C; 12, galectin-12; DFS, disease free survival; DSS, disease specific survival; HR; Hazard Ratio; OS, overall survival; RFS, relapse free survival.

Supplementary Table S1: GEO Datasets of HNSCC used in TISCH2 analysis.

Dataset Name	Patients	Cells from GEO and Array Express Databases	Platform	Primary Tumor or Metastasis	PMID
HNSC_GSE103322	18	5,902	Smart-seq2	Primary	29198524
HNSC_GSE139324	26	130,721	10x Genomics	Primary	31924475
LSCC_GSE150321	2	16,869	10x Genomics	Primary	32638385
NPC_GSE150430	15	45,959	10x Genomics	Primary	32686767
NPC_GSE162025	10	176,44	10x Genomics	Primary	33531485
OSCC_GSE172577	6	57,503	10x Genomics	Primary	34044317
HNSC_GSE180268	6	53,66	10x Genomics	Primary, Metastatic	34471285

Legend: HNSC, head and neck squamous cell carcinoma; LSCC, laryngeal squamous cell carcinoma; NPC, nasopharyngeal carcinoma; OSCC, oropharyngeal squamous cell carcinoma; PMID, PubMed Unique Identifier.

Supplementary Table S2: Mutations of galectins in cBioPortal.

Galectin	Protein change	Mutation type	Copy
LGALS1	G104V	Missense	Gain
	K29N	Missense	Shallow Deletion
	R74W	Missense	N/A
	G15*	Nonsense	Gain
LGALS3	Q20*	Nonsense	Shallow Deletion
LGALS3BP	S557F	Missense	Diploid
	D549N	Missense	Diploid
	S512L	Missense	Diploid
	Q422E	Missense	Diploid
LGALS4	E259K	Missense	Diploid
	T78M	Missense	Diploid
LGALS8	D205E	Missense	Gain
LGALS9	G69R	Missense	N/A
	L11P	Missense	Gain
LGALS9B	S6C	Missense	Diploid
	S12N	Missense	Diploid
	P64H	Missense	Shallow Deletion
	Q94K	Missense	Diploid
	G176V	Missense	Diploid
	W287L	Missense	Diploid
	W288L	Missense	Diploid
	K297E	Missense	Diploid
LGALS9C	P150S	Missense	Shallow Deletion
	W288L	Missense	Diploid
	Y331C	Missense	N/A
	V184M	Missense	N/A
LGALS12	PRSS23-LGALS12 Fusion	Fusion	Amplification
	P45R	Missense	N/A
	E144K	Missense	Shallow Deletion
	Y195C	Missense	Gain
	Y196C	Missense	Gain
	E288K	Missense	Diploid

Legend: N/A, non-available information.
